# Prognostic utility of the lactate-to-albumin ratio for predicting 28-day all-cause mortality in critically ill cases with acute sepsis: A retrospective study on the basis of MIMIC-IV critical care database

**DOI:** 10.5937/jomb0-59662

**Published:** 2026-01-06

**Authors:** Jiaqi Cheng, Jiatong Hou, Yuefu Wang

**Affiliations:** 1 Beijing Shijitan Hospital, Affiliated to Capital Medical University, Beijing, China

**Keywords:** sepsis, lactate-to-albumin ratio, all-cause mortality, MIMIC-IV database, sepsa, odnos laktata i albumina, smrtnost od svih uzroka, baza podataka MIMIC-IV

## Abstract

**Background:**

Sepsis constitutes a systemic dysregulated host response to infection and remains a predominant cause of ICU mortality globally. Given the limitations of conventional prognostic models (e.g., SOFA and APACHE II), incorporating variably subjective parameters, there is a pressing need to identify robust, objective biomarkers for early mortality risk stratification. This investigation delineated the prognostic significance of the lactate-to-albumin ratio (LAR) in predicting 28-day all-cause mortality (28-DACM) among critically ill septic cases.

**Methods:**

We performed a retrospective analysis utilizing the MIMIC-IV database (2008-2019), comprising 5,398 adult cases who met Sepsis-3 diagnostic criteria. Clinical and laboratory data within the initial 24-h post-ICU admission were extracted. The LASSO regression algorithm was implemented as a regularization technique to mitigate multicollinearity, enhance model generalizability, and facilitate high-dimensional feature selection. It was made to evaluate the prognostic utility of LAR through Kaplan-Meier (KM) survival estimation, receiver operating characteristic (ROC) curve analysis, and multivariate logistic regression modeling.

**Results:**

LAR values were remarkably escalated in non-survivors relative to survivors (median, 0.9 vs. 0.6; P &lt; 0.001). ROC curve analysis unveiled that LAR outperformed lactate (AUC: 63.52% ), albumin (AUC: 43.34% ), and the SOFA score (AUC: 59.87% ), achieving the highest discriminatory capacity (AUC: 64.71% ; 95% CI: 62.85-66.58%). An optimal LAR threshold of 1.032 was identified, attaining sensitivity and specificity of 45.1% and 76.6% , respectively. KM analysis uncovered remarkably attenuated 28-day survival in cases with LAR &gt;1.032 (P &lt; 0.001). Multivariate logistic regression confirmed LAR as an independent predictor of 28-DACM (OR = 1.32; P &lt; 0.001), following adjusting for confounding variables.

**Conclusions:**

The LAR serves as a clinically accessible, objective biomarker with superior prognostic performance relative to established indicators in association with sepsis. Its integration into early risk assessment algorithms may enhance prognostication and inform timely therapeutic decision-making. Prospective, multicenter investigations are warranted to validate its external generalizability and clinical utility.

## Abstract (sr)

**Uvod:**

Sepsa predstavlja sistemski disregulisani odgovor domacina na infekciju i ostaje dominantan uzrok smrtnosti na intenzivnoj nezi širom sveta. S obzirom na ograničenja konvencionalnih prognostičkih modela (npr. SOFA i APACHE II), koji uključuju varijabilno subjektivne parametre, postoji hitna potreba za identifikacijom robusnih, objektivnih biomarkera za ranu stratifikaciju rizika od smrt-nosti. Ovo istraživanje je definisalo prognostički značaj odnosa laktata i albumina (LAR) u predviđanju 28-dnevnog mortaliteta od svih uzroka (28-DACM) kod kritično bolesnih septičnih slučajeva.

**Metode:**

Sproveli smo retrospektivnu analizu koristeci bazu podataka MIMIC-IV (2008-2019), koja je obuhvatila 5.398 odraslih slučajeva koji su ispunjavali dijagnostičke kriterijume za sepsu 3. Ekstrahovani su klinički i laboratori-jski podaci u prvih 24 sata nakon prijema na intenzivnu negu. LASSO algoritam regresije je implementiran kao tehnika regularizacije radi ublažavanja multikolinearnosti, poboljšanja generalizacije modela i olakšavanja odabira visokodimenzionalnih karakteristika. Napravljen je da bi se procenila prognostička korisnost LAR-a putem procene preživljavanja Kaplan-Majerovom (KM ), analize ROC krive i multivarijantnog logističkog regresionog modeliranja.

**Rezultati:**

Vrednosti LAR-a su bile značajno povecane kod nepreživelih u odnosu na preživele (m edijana, 0,9 naspram 0,6; P &lt; 0,001). Analiza ROC krive je otkrila da je LAR nadmašio laktat (AUC: 63,52% ), albumin (AUC: 43,34% ) i SOFA skor (AUC: 59,87% ), postižući najveći diskriminatorni kapacitet (AUC: 64,71% ; 95% CI: 62,85-66,58%). Identifikovan je optimalni prag LAR-a od 1,032, uz dostižanje osetljivosti i specifičnosti od 45,1% i 76,6% , respektivno. KM analiza je otkrila značajno smanjeno preživljavanje od 28 dana u slučajevima sa LAR &gt;1,032 (P &lt; 0,001). Multivarijantna logistička regresija je potvrdila LAR kao nezavisni prediktor 28-DACM (OR = 1,32; P &lt; 0,001), nakon prilagođavanja za zbunjujuće varijable.

**Zaključak:**

LAR služi kao klinički pristupačan, objektivan biomarker sa superiornim prognostičkim učinkom u odno-su na utvrđene indikatore povezane sa sepsom. Njegova integracija u algoritme za ranu procenu rizika može poboljšati prognozu i informisati blagovremeno donošenje terapijskih odluka. Potrebna su prospektivna, multi-centrična istraživanja kako bi se potvrdila njegova eksterna generalizacija i klinička korisnost.

## Introduction

Sepsis constitutes a multifaceted clinical syndrome recognized by a maladaptive and dysregulated host immune response to infection, manifesting through physiological, pathological, and biochemical perturbations that collectively disrupt systemic homeostasis [1]. Over recent decades, advances in timely antibiotic administration, fluid resuscitation, and multi-organ support have led to a gradual decline in sepsis-related mortality. However, mortality rates remain unacceptably high, highlighting the need for further improvement. The most recent Global Burden of Disease report estimated that in 2017, sepsis globally influenced approximately 48.9 million cases and was linked to a mortality rate of 22.5%, contributing to nearly 20% of all deaths globally [2] [3]. Sepsis frequently leads to varying degrees of organ dysfunction and demands urgent medical intervention, and without prompt and effective treatment, mortality rates can exceed 30-35% [4]. In addition to its clinical severity, septic shock imposes a significant economic burden, with annual healthcare costs estimated at $24 billion [5]. Clinical evidence suggests that early identification of sepsis, and timely, targeted management of prognostic risk factors can significantly reduce mortality [6]. Consequently, early assessment of disease severity is critical in improving outcomes for patients with sepsis.

In the intensive care unit (ICU), the Sequential Organ Failure Assessment (SOFA) and Acute Physiology and Chronic Health Evaluation II (APACHE II) scoring systems are routinely employed as integrative tools to quantify illness severity and prognosticate clinical outcomes. While both frameworks are grounded in robust physiological and clinical parameters, their reliance on a multitude of variables, time-intensive data collection, and the incorporation of semi-subjective assessments can constrain their applicability in urgent, high-acuity contexts where rapid decision-making is critical. Lactate, an intermediary metabolite generated during anaerobic glycolysis, has emerged as a surrogate biomarker for systemic hypoperfusion and cellular hypoxia, exhibiting a strong linkage with multiorgan dysfunction and adverse outcomes in critically ill populations. Nevertheless, its diagnostic and prognostic fidelity in sepsis is attenuated by a range of extrinsic and intrinsic modifiers, involving hepatic insufficiency, aberrations in proteolytic pathways, and exogenous agents, such as metformin, collectively compromising its specificity and limiting its standalone clinical utility [7] [8] [9].

Similarly, serum albumin has been shown to predict outcomes in septic patients [10]; however, its levels are significantly affected by nutritional status. Patients with malignancies or malnutrition frequently exhibit hypoalbuminemia, limiting the prognostic reliability of albumin alone. To overcome these limitations, recent investigations have proposed the LAR as a potentially more robust and integrative biomarker for mortality risk stratification in septic patients, reflecting a composite measure of both metabolic stress and nutritional/inflammatory status. Despite the initial promise demonstrated by these studies, their generalizability remains limited due to small cohort sizes, methodological inconsistencies, and the absence of a standardized LAR threshold for prognostic delineation. Consequently, the present study was designed to rigorously evaluate the predictive capacity of LAR for 28-day all-cause mortality (28-DACM) in cases with sepsis. Data for this investigation were retrospectively acquired from the Medical Information Mart for Intensive Care IV, version 2.0 (MIMIC-IV) database, comprising a comprehensive repository of ICU admissions recorded between 2008 and 2019.

## Materials and methods

### Database introduction

The dataset utilized was attained from the MIMIC-IV, particularly version 2.0, an open-access, publicly available clinical database curated by a research team affiliated with the MIT [11] [12]. This repository comprises detailed, longitudinal clinical data from patients admitted to a single tertiary-care ICU between 2008 and 2019, comprising demographic information, physiological measurements, laboratory and imaging outputs, therapeutic interventions, complications, and diagnostic codes. MIMIC-IV is broadly acknowledged as a validated resource in critical care research. To uphold patient privacy and data security, all personal identifiers were removed and substituted with anonymized, randomly generated codes. As the dataset is fully de-identified in compliance with the HIPAA standards, individual informed consent and additional institutional ethical review were waived. Access to the database was formally granted under project approval number of 50988851.

### Population selection criteria

**Figure 1:**
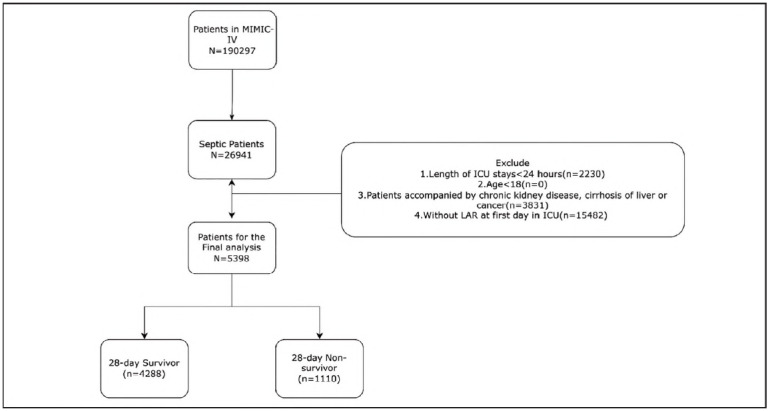
Flowchart of the study selection process.

The study population was identified based on the Sepsis-3 definition (1), including adult patients diagnosed with sepsis during their initial ICU admission. Serum lactate and albumin concentrations obtained within the initial 24 h of ICU admission were extracted for analysis. Patients were stratified into two cohorts according to their 28-day survival status. Utilizing the Sepsis-3 diagnostic criteria, an initial cohort of 26,941 septic patients was identified from the MIMIC-IV database. Inclusion criteria involved: (1) age 218 years; (2) first ICU admission during the indexed hospital stay; and (3) a confirmed diagnosis of sepsis. Patients' exclusion was on the basis of the following conditions: (1) length of stay in ICU < 24 h; (2) documented history of malignancy or hepatic pathology; and (3) incomplete clinical records, specifically missing lactate or albumin values. After rigorous application of these criteria, 5,398 patients were totally regarded eligible and involved in the final cohort. The detailed flow of the patient selection is depicted in Figure 1.

### Data extraction

The entirety of the dataset employed in this investigation was acquired from the MIMIC-IV database. Variables retrieved involved a comprehensive spectrum of patient-level data, comprising demographic attributes (age, sex, race), hemodynamic and respiratory parameters, biochemical indices (serum lactate and albumin concentrations), and validated critical illness severity metrics, namely the SOFA and SAPS II. Additionally, information on clinical interventions, involving mechanical ventilation and continuous renal replacement therapy, and documented comorbidities at ICU admission (including but not limited to hypertension, coronary artery disease, and diabetes mellitus) were incorporated, in which comorbidity identification was facilitated via corresponding ICD coding schemas. The principal variable, the LAR, was operationalized as the quotient of serum lactate (expressed in mmol/L) divided by serum albumin (expressed in g/dL). All laboratory values were restricted to those obtained within the initial 24-h window post-ICU admission to capture the acute physiological status. Data extraction procedures were executed through PostgreSQL-based structured query language (SQL) commands, utilizing standardized scripts available through the publicly accessible repository, thereby ensuring reproducibility and methodological transparency.

### Grouping and endpoint events

Patients were stratified into dichotomous cohorts on the basis of their survival status at 28 days post-ICU admission: the survival cohort (n = 4,288) and the non-survival cohort (n = 1,110). The allcause mortality during the 28-day interval following ICU admission was the primary outcome measure. The 28-DACM rate was quantified as the ratio of cumulative deaths during this observation window to the average at-risk population, thereby providing a temporal incidence proportion reflective of short-term prognostic burden.

### Statistical analysis

Statistical analyses were performed using R 4.3.2 and SPSS 22.0. The Kolmogorov-Smirnov test was employed to rigorously figure out continuous variables' normality assumption. Variables conforming to parametric distributions were reported as mean ± standard deviation (SD), whereas those violating normality were expressed as median accompanied by interquartile ranges (IQR). Notably, categorical variables were delineated by absolute frequency and relative percentage. Intergroup comparisons of continuous variables were performed using independent samples t-tests or one-way ANOVA, as appropriate based on data distribution and the number of groups compared, while distributional differences in categorical variables were assessed using Pearson's Chi-square test or Fisher's exact test, as appropriate. To circumvent multicollinearity and facilitate model development, the least absolute shrinkage and selection operator (LASSO) regression was implemented; this penalization method applies L1 regularization to constrict regression coefficients, effectively excluding superfluous predictors and preserving only those with maximal prognostic import. Discriminatory performance of lactate, albumin, LAR, and SOFA scores in predicting 28-DACM was quantified via receiver operating characteristic (ROC) curve analysis, with sensitivity, specificity, and area under the curve (AUC) values computed accordingly. The optimal threshold for LAR was identified through maximization of the Youden index, facilitating dichotomization of the cohort into low- and high-LAR strata. Comparative survival analyses between these strata were conducted employing KM estimators, and significance's assessment was through log-rank testing. Denoting statistical significance could be symbolized as P below 0.05.

## Results

### Baseline demographic and clinical characteristics

The baseline characteristics of patients in the 28-day survival and non-survival groups are presented in Table 1. In this investigation, 5,398 patients totally met the inclusion criteria, comprising 3,032 men (56.2%) and 2,366 women (43.8%), with a median age of 64.9 (IQR: 53-77) years. The overall 28-DACM rate was 20.6%.

**Table 1:** Comparison of baseline characteristics between survivors and non-survivors.

Variable	Total <br>(N = 5398)	28-day survivors<br>(n=4288)	28-day non-survivors<br>(n = 1110)	P-value
Age (yr)	64.9 (53,77)	63.7 (52,76)	69.4 (58,81)	<0.001
Gender (%)				0.974
Female	2366 (43.8)	1879 (43.8)	487 (43.9)	
Male	3032 (56.2)	2409 (56.2)	623 (56.1)	
Race (%)				0.002
White	3248 (60.2)	2626 (61.2)	622 (56)	
Others	2150 (39.8)	1662 (38.8)	488 (42)	
HR (beat/min)	89.4 (77.5,102.4)	88.9 (77.4,101.2)	91.3 (77.7,105.1)	0.001
MBP (mmHg)	75.2 (69.4,82.3)	75.7 (70.1,82.7)	73.4 (67.2,80.3)	<0.001
Temperature (°C)	36.9 (36.6,37.3)	36.9 (36.6,37,4)	36.8 (36.3,37.2)	<0.001
SO2 (%)	97.3 (95.7,98.7)	97.4 (95.9,98.7)	96.9 (95.2,98.5)	<0.001
HTN (%)				<0.001
Yes	976 (18.1)	843 (19.7)	133 (12)	
No	4422 (81.9)	3445 (80.3)	977 (88)	
CHD (%)				<0.001
Yes	587 (10.9)	512 (11.9)	75 (6.8)	
No	4811 (89.1)	3776 (88.1)	1035 (93.2)	
Diabetes (%)				<0.001
Yes	605 (11.2)	540 (12.6)	65 (5.9)	
No	4793 (88.8)	3748 (87.4)	1045 (94.1)	
AKI (%)				0.136
Yes	1063 (19.6)	862 (20.1)	201 (18.1)	
No	4355 (80.4)	3426 (79.9)	909 (81.9)	
CRRT (%)				<0.001
Yes	719 (13.3)	454 (10.6)	265 (23.9)	
No	4679 (86,7)	3834 (89.4)	845 (76.1)	
Ventilation (%)				<0.001
Yes	3578 (66.3)	2677 (62.4)	901 (81.2)	
No	1820 (33.7)	1611 (37.6)	209 (18.8)	
SOFA	3 (2,5)	3 (2,5)	4 (3,6)	<0.001
SAPSii	41 (32,52)	39 (30,48)	53 (42,64)	<0.001
Alb (g/dL)	30 (25,30)	30 (26,35)	29 (23,34)	<0.001
Lac (mmol/L)	1.9 (1.3,3.1)	1.8 (1.2,1.8)	2.5 (1.6,4.5)	<0.001
LAR	0.6 (0.4,1.1)	0.6 (0.4,1.0)	0.9 (0.5,1.7)	<0.001

Relative to the survival cohort, cases in the non-survival group were significantly older and exhibited remarkably elevated heart rates (P < 0.001). Concomitantly, they demonstrated reduced mean arterial pressure, peripheral oxygen saturation, and core body temperature (P < 0.001), indicative of hemodynamic and physiological compromise. Notably, the prevalence of chronic comorbid conditions, specifically hypertension, diabetes mellitus, and coronary artery disease, was noticeably lowered in the non-survival group (P < 0.001), reflecting that acute disease severity rather than baseline morbidity could be the predominant determinant of outcome in this cohort. Conversely, utilization rates of critical life-sustaining interventions, involving invasive mechanical ventilation and continuous renal replacement therapy (CRRT), were disproportionately higher among non-survivors, reflecting a greater degree of clinical deterioration. Moreover, non-survivors exhibited remarkably escalated scores on both the SOFA and SAPS II at ICU admission, emphasizing the escalated severity of systemic dysfunction in this group. Biochemical profiling further revealed significantly diminished serum albumin concentration and elevated lactate level (P < 0.001), culminating in a notably escalated LAR among non-survivors relative to their surviving counterparts (P < 0.001). Notably, the absence of significant difference especially in the incidence of acute kidney injury was noteworthy between the two principal groups.

### LAR as an independent factor for all-cause mortality on day 28 of hospitalization

**Figure 2:**
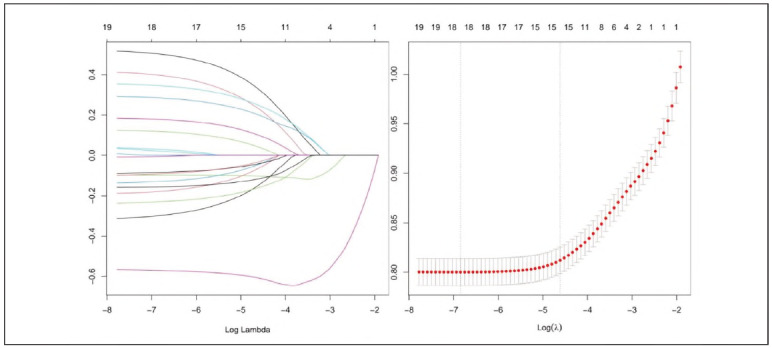
Lasso regression-based variable selection. A. Variations of variable coefficients; B. The process of selecting the optimal value of the parameter λ in the lasso regression model carried out by the cross-validation method.

Following potential variables' screening through Lasso regression, 16 variables suspected to exhibit linkage with 28-DACM in cases with sepsis were involved in the Lasso regression analysis. The coefficient profiles of the variables are presented in Figure 2A, Figure 2B. A 10-fold cross-validation method was applied iteratively. The results revealed that the variables significantly linked to 28-DACM in septic patients were age, ethnicity, mean arterial pressure, body temperature, blood oxygen level, LAR, administration of CRRT, and mechanical ventilation, along with comorbidities, such as hypertension, chronic renal insufficiency, and diabetes mellitus.

### ROC curve analysis and KM curves

**Figure 3:**
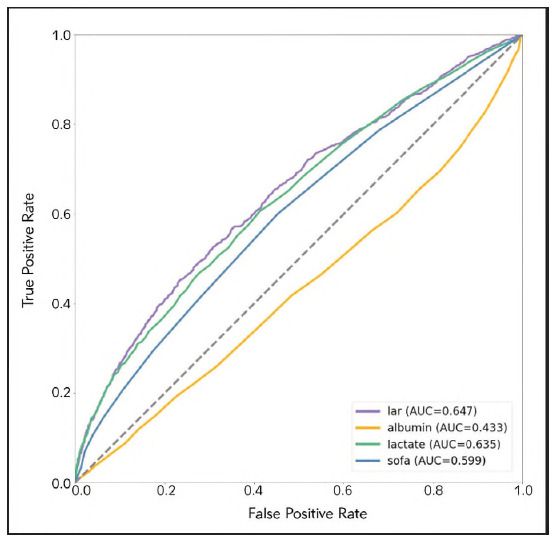
ROC curves of 4 indicators for predicting in-hospital mortality. The purple solid line indicates the ROC curve of the LAR. The yellow solid line indicates the ROC curve for Albumin. The green solid line indicates the ROC curve for Lactate. The blue indicates the ROC curve of SOFA. LAR, lactate/albumin ratio; SOFA, Sequential Organ Failure Assessment.

**Figure 4:**
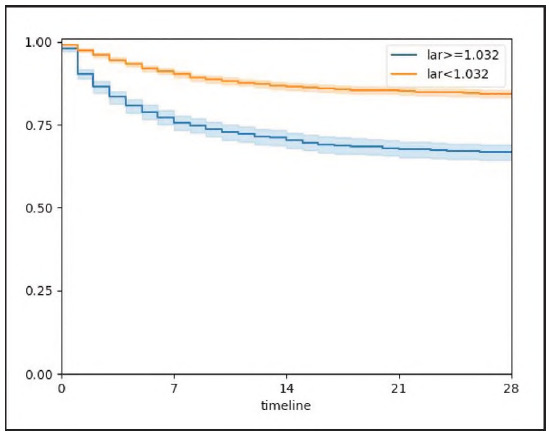
Kaplan-Meier survival analysis curves for all-cause mortality within 28-d of hospital admission.

ROC curve analysis was employed to figure out and compare the prognostic efficacy of the LAR, serum lactate, serum albumin, and SOFA scores in predicting 28-DACM among cases with sepsis. The corresponding ROC metrics, detailed in Table 2 and visualized in Figure 3, reflect the discriminative performance of these indices. The LAR yielded an AUC of 64.72% (95% CI: 62.85-66.58%), surpassing that of lactate alone (AUC: 63.52%, 95% CI: 61.6465.39%) and demonstrating non-inferiority to the SOFA score (AUC: 59.87%, 95% CI: 57.9761.77%). Furthermore, LAR exhibited remarkably superior prognostic capacity relative to serum albumin, which demonstrated limited utility (AUC: 43.34%, 95% CI: 41.37-45.32%). These outcomes reflect the utility of LAR as a robust, objective biomarker with meaningful prognostic implications in the septic population. The optimal threshold for LAR, as determined via maximization of Youden's index, was identified at 1.032. At this cutoff, LAR demonstrated sensitivity and specificity of 45.1% and 76.6%, respectively. All enrolled patients were divided into low LAR group (LAR < 1.032, n = 3,889) and high LAR group (LAR 2 1.032, n = 1,506). KM survival analysis unveiled a significantly escalated cumulative mortality in the high LAR group relative to the low LAR group (P < 0.001), further substantiating the prognostic stratification utility of LAR particularly for sepsis-associated critical illness (Figure 4, P < 0.001).

**Table 2:** Summary of ROC curve metrics corresponding to Figure 3.

Variables	AUC	95%CI	Threshold	Sensitivity	Specificity
LAR		62.85% - 66.58%	1.032	0.451	0.766
Lactate	63.52%	61.64% - 65.39%	2.05	0.606	0.587
Albumin	43.34%	41.37% - 45.32%	4.95	0.006	0.999
SOFA	59.87%	57.97% - 61.77%	3.5	0.600	0.548

## Discussion

The outcomes of this retrospective investigation, derived from the comprehensive MIMIC-IV database, highlighted the LAR as an autonomous and potent prognostic biomarker for 28-DACM in cases with sepsis. Quantitative assessment through ROC curve analysis unveiled that LAR attained a superior discriminative capacity, reflected by an AUC of 64.71%, exceeding that of serum lactate (63.52%) and albumin (43.34%) when evaluated independently, and notably outperforming the widely implemented SOFA score, which yielded an AUC of 59.87%. Furthermore, stratification based on the empirically derived LAR threshold of 1.032 delineated distinct survival trajectories, with KM analysis demonstrating a statistically significant elevation in 28-DACM among patients exhibiting LAR values 2 1.032 relative to their lower LAR counterparts (P < 0.001).

For decades, researchers have attempted to find out reliable tools to predict the severity and prognosis of sepsis. Traditional scoring systems, involving SOFA, APACHE II, and SIRS have been widely adopted; however, these tools involve multiple variables and complex calculations, limiting their practicality in urgent clinical settings. In recent years, LAR has emerged as a novel prognostic biomarker for various critical diseases, including pancreatitis, acute respiratory failure, and heart failure, demonstrating efficacy in predicting mortality [13] [14]. Nevertheless, large-scale studies investigating the prognostic value of LAR in sepsis have remained limited.

Lactate is a well-established marker of tissue hypoxia, perfusion deficits, and metabolic dysfunction. Consistent with previous findings [15], this study confirmed that the elevated lactate level is linked to poor outcomes in septic patients. However, interpreting serum lactate level can be challenging due to potential confounding factors. Patients with hepatic dysfunction may exhibit impaired lactate clearance, and medications, such as salbutamol and metformin, can induce lactate elevation, although such effects were not evident in this cohort. Moreover, certain critically ill patients may present with low venous lactate concentrations, thereby limiting the prognostic reliability of lactate as a standalone biomarker [16] [17].

The systemic inflammatory response in sepsis results in the release of abundant pro-inflammatory mediators, which can increase vascular permeability, leading to hypoproteinemia and a reduction in effective circulating blood volume [18]. Consequently, hypoalbuminemia may be linked to adverse outcomes in patients with sepsis. Previous studies have identified serum albumin as a strong predictor of mortality, particularly in elderly septic patients [19]. However, albumin level may be influenced by chronic diseases, nutritional status, and other non-inflammatory factors, thereby limiting its reliability as a standalone prognostic marker. To address these limitations, this study utilized the LAR, combining two biomarkers that reflect different pathophysiological processes. The objective was to enhance predictive accuracy by compensating for the limitations of individual markers, as lactate and albumin typically exhibit inverse changes during critical diseases [20].

Previous studies by Cakir and Turan [21] and Shin et al. [22] independently demonstrated that LAR was a superior predictor of in-hospital mortality compared with either lactate or albumin alone. These findings align with the present results derived from the MIMIC-IV database, further supporting the clinical utility of LAR in prognostication. Routine monitoring of LAR may therefore aid in the early identification and management of high-risk septic patients. Two recent small-scale studies examining the prognostic value of LAR in sepsis reported optimal cutoff values of 0.16 (based on different albumin units) and 1.1, respectively [23] [24], which are broadly consistent with the threshold identified in this study. Despite its strengths, this study's numerous limitations are noteworthy. Firstly, as a single-center retrospective cohort study, the findings are inherently limited by potential biases and confounding factors, and no causal relationships could be definitively established. Secondly, the analysis was based solely on LAR values obtained during the first 24 h of ICU admission, hindering assessment of dynamic changes over time and their prognostic implications. Thirdly, the data were drawn from the MIMIC-IV database, spanning patient admissions from 2008 to 2019. Over such a long period, advances in medical care and evolving treatment protocols might introduce heterogeneity, potentially influencing the generalizability of the results.

## Conclusions

This investigation delineated the LAR as an independent and robust prognostic biomarker for 28-DACM in cases with sepsis. The LAR demonstrated superior discriminative capacity relative to either serum lactate or albumin individually, while exhibiting prognostic capability comparable to that of the SOFA score. These findings implicate LAR as a readily quantifiable, objective parameter with significant potential to enhance early risk stratification frameworks and optimize clinical decision-making processes in the sepsis treatment paradigm. Nonetheless, This study is a retrospective analysis and only involved a single set of LAR data, which does not allow for dynamic observation of LAR trends across different patients. comprehensive validation through rigorously designed, large-scale, multicenter prospective cohorts remains indispensable to substantiate its prognostic fidelity and to promote its integration into standardized clinical practice guidelines.

## Dodatak

### Conflict of interest statement
